# Chatbot for communicating with university students in emergency situation

**DOI:** 10.1016/j.heliyon.2023.e19517

**Published:** 2023-08-30

**Authors:** Antonio Balderas, Roberto Fermín García-Mena, Milagros Huerta, Nestor Mora, Juan Manuel Dodero

**Affiliations:** a Departamento de Ingeniería Informática, Escuela Superior de Ingeniería, Puerto Real, 11519, Cádiz, Spain; b Departamento Ingeniería Mecánica y Diseño Industrial, Escuela Superior de Ingeniería, Puerto Real, 11519, Cádiz, Spain; c Departamento Ingeniería en Automática, Electrónica, Arquitectura y Redes de Computadores, Escuela Superior de Ingeniería, Puerto Real, 11519, Cádiz, Spain

**Keywords:** 0000, 1111, Chatbot, Students, Emergency situation, COVID-19

## Abstract

Chatbots have arrived in higher education, and professors are trying to make the most of them. Typically, chatbots are used to help students learn academic subjects. In times of crisis, such as the COVID-19 pandemic, students who were not living with their families during the course, especially international students, were isolated and in critical situations. The student services offices were in constant contact with these students to solve problems, advise them and support them during their stay, within the constraints of confinement and the guidelines dictated by the country at the time. The student services offices were overwhelmed trying to help these students because, although the students' problems were very recurrent, the government guidelines changed from one day to the next. This article proposes the use of a chatbot to provide initial support to students during crisis situations, and facilitate communication between them and the university. The chatbot was tested by more than 160 students and student services staff. The findings support the use of chatbots as a potential tool to facilitate communication with students in emerging emergency situations, and encourage universities to adopt these types of smart tools to be prepared to respond quickly and efficiently to students in times of crisis.

## Highlights

• COVID-19 particularly affected university students living away from their home country.
• In the event of an emergency, student services must provide students with continuously updated information.
• This research proposes a chatbot to facilitate communication between students and student services in emergency situations.
• Chatbot enables adaptation to regulatory changes in emergency situations.
• Both students and university staff gave the chatbot a positive evaluation.

## 1 Introduction

The COVID-19 crisis demonstrated that higher education institutions were not digitally prepared to respond to the needs of their students [1]. Therefore, the pandemic and confinement has been taken advantage of by universities to carry out a necessary digital transformation, expanding the catalogue of tools and methodologies to support blended learning [2].

However, the problem of maintaining teaching in higher education was not the only one that needed to be addressed for students. The COVID-19 crisis and related measures resulted in negative effects on students' social networks and mental health [3]. Students, as well as general citizens, were faced with an unfamiliar situation in which there were specific prohibitions to comply with due to the extraordinary measures. A particularly problematic case was that of students who were not living at their family house during the course, especially international students, as they were not close to their family and friends, and could be much more affected by not having the necessary social support to cope with the stressful situation [4]. Universities made efforts to communicate with and help these students through message groups, telephone support, etc. The student services offices were very busy as the borders were closed and these students could not even consider returning home and the universities had to support them. These staff would have needed some kind of tool to deal with these students, because although many of the students' issues were similar, government guidelines on what could and could not be done changed from one day to the next, and students needed to be kept up to date.

This research proposes the use of a chatbot to facilitate communication with student services offices and support students in emergency situations in the future. Chatbots are technological artefacts that interact with users through natural language, and are generally designed to provide information to users in a specific area. Chatbots were adopted after the COVID-19 pandemic to support students' autonomous learning [5], [6]. Our chatbot has been implemented using Google's DialogFlow technology and has been tested by both the staff of the student services offices from six European universities and students from a university in southern Spain. In order to be prepared for a new emergency situation, the aim of this work is to assess students' perceptions of the use of chatbots as a means of communicating with student services in such a situation.

This research is conducted within the Research for Innovative Practices in Emergency Management of Erasmus (RIPEC) project. The project aims to compile the experiences of university staff and students in order to identify the main issues and identify innovative solutions for Erasmus mobility in case of a new crisis.

The rest of the article is organised as follows. Next, the background is presented. Then, the technical details of the chatbot are presented. Fourth, the results of the evaluation are presented. Finally, it concludes with a discussion and the conclusions of the research.

## 2 Background

Virtual applications were essential during the pandemic, allowing society to continue its activities in many sectors despite the lockdown and subsequent distancing measures. In this sense, the higher education sector was able to continue its activities, rapidly adapting its infrastructures in order to continue teaching in the best possible conditions. However, aspects such as support to students who live away from home in emergency situations may have been neglected, with student services offices having to assist these students by providing information and support in their particular circumstances. Students had to cope with numerous uncertainties, including whether they would be able to return home given the restrictions on travelling, whether they could leave the accommodation to shop or eat, or even whether they could go to the doctor. In addition, they suffered the psychological effects of being isolated from other people, compounded by the fact that they had no family nearby. Student services offices were overwhelmed by the number and variety of enquiries they received from students during the emergency. This background is divided into two sections; firstly, we present research that uses technology to support students in emergency situations. Secondly, we present research that uses chatbots to support students in higher education.

### 2.1 Student support software for emergencies

Higher education institutions have been able to continue operating because they mostly followed a blended learning approach, with a virtual learning environment (VLE) for their courses [7]. VLE provide teaching staff with numerous tools to communicate with students, such as forums, portfolios, chats or the messaging system of the virtual campus itself. These tools supported the continuation of higher education, although they were mainly used for academic subjects and students were negative about their lack of interactivity [8].

One of the tools that provide more interactive communication, and whose use has grown significantly since lockdown, is video conferencing [9]. These integrate seamlessly with virtual courses and allow teachers to continue with their classes [10]. In this study, psychology students used videoconferencing to conduct psychotherapy during the lockdown, demonstrating that this tool can be used to communicate and treat their patients [11]. However, the literature also suggests that videoconferencing can cause anxiety in students. [12], and in general students are reluctant to connect their camera when they are off campus. [13]. Another emergency scenario in which video conferencing has been used to help people can be found in the context of the invasion of Ukraine. In this work, video conferencing has been used to assist in surgical operations [14].

The problem faced by the student services offices was the difficulty in handling communications, finding an adequate and timely response to the students who needed it, some of them with specific problems, others common to many. One of the solutions was to use broadcasting groups in applications such as Whatsapp or Telegram [15]. This study defined profiles in the use of social networks by international students during the period of confinement and claimed that students facing psychological challenges communicate more online than others [16].

A tool that allows communication with a large number of students in a scalable and usable way through a communication channel such as Telegram is a chatbot. In the next sub-section we will explore approaches for using chatbots to help students.

### 2.2 Chatbots to assist students

There is already research in educational technology that has used chatbots in higher education subjects, with satisfactory results in helping students improve their academic performance [17], [18], [5]. Students who have been able to use a chatbot in some of their subjects are discovering the benefits of chatbots and are requesting that they be made available in other aspects of their academic life [19].

Along the same lines, other work has demonstrated the effectiveness of chatbots in helping people communicate in difficult situations. CapacitaBOT is an educational chatbot that aims to help users with intellectual disabilities to understand, control and improve their social skills [20]. SPeCECA is a first aid chatbot designed to help people respond well to health emergencies [21]. This research shows how the processes of empathy and resilience through the interaction between the student and the chatbot could help to cope with the COVID-19 disorder [22].

COVID-19 has also led to a growth in the use of chatbots to support students. The students in Kohnke's research indicated that being able to interact with a chatbot reduced their sense of isolation and had a positive impact on their learning [23]. The work presented by Tiwari et al. used a chatbot to answer questions about COVID-19 [24], while the chatbot introduced by Kim and Riio was used to promote social distancing [25].

Unfortunately, the implementation details of chatbots are not published and most are based on a series of questions and answers, similar to a list of frequently asked questions (FAQ), and can hardly provide the context of a conversation similar to the one we would have with a human.

## 3 Chatbot

The chatbot to assist students in emergency situations is developed within the framework of the RIPEC project. This section first describes the architecture of the system that comprises the chatbot. Secondly, it presents the implementation of the assistant to converse and provide the appropriate responses. Thirdly, the configuration of the chatbot memory system is shown.

### 3.1 System architecture

The chatbot was designed and built using Dialogflow [26]. Dialogflow is a platform developed by Google that makes it easy to build conversational bots. It provides a pre-configured platform and interface, as well as easy integration with other services. The chatbot is available in a GitHub repository.^1^

Two interfaces were implemented for the chatbot: a chat integrated into the RIPEC website and another one using Telegram. *Dialogflow Messenger* was utilized to incorporate the chatbot into the website, which offers an HTML code that needs to be inserted into the website's code. This code generates a floating button that, when clicked, opens a chat window. As for the Telegram bot, it was created using a separate bot called *BotFather*. This bot generates a unique token that identifies the new bot. The token should be entered in a text box within the Dialogflow integrations screen.

**Figure 1:**
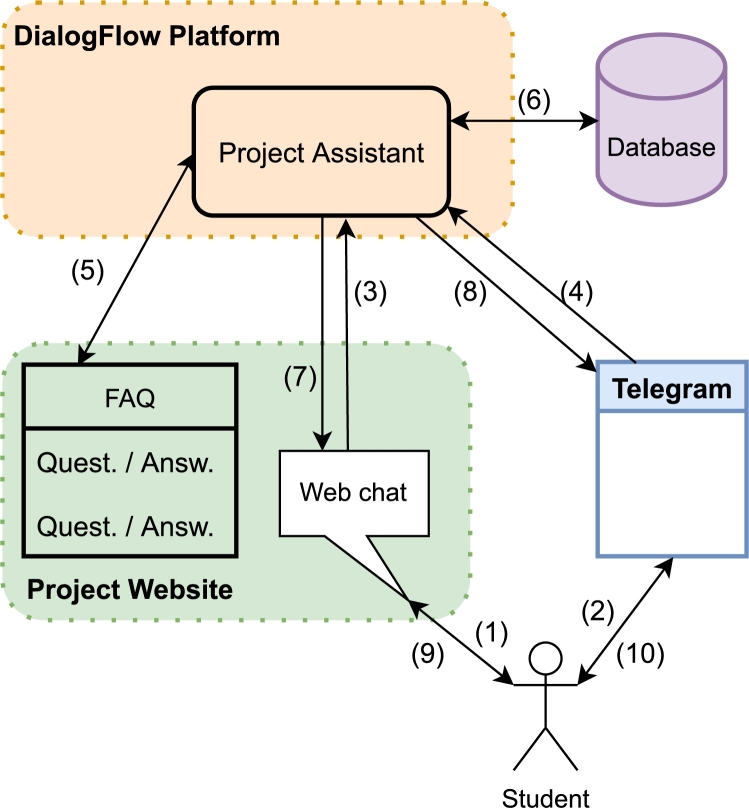
Architecture of the components integrated in the design of the chatbot.

The architecture of the system that builds the chatbot is shown in Fig. 1. This figure shows how the student will be able to talk to the chatbot via the chat enabled on the project website (1) or via the instant messaging application Telegram (2). The student's message is then sent to be processed by the DialogFlow assistant, regardless of whether the message comes from the website chat (3) or the Telegram chat (4). The assistant regularly updates its knowledge base entries via the FAQ entries on the project website (5). The FAQ contains general information that would be of interest to any student, regardless of their university. If the student's question can be answered by its knowledge base, it will send the answer via its source channel (7) or (8). On the other hand, if it does not find an answer in its knowledge base, it will try to manage the answers through the information contained in the database (6). The information in this database is specific to each university, so the chatbot will have asked the student's university beforehand to provide the appropriate answer. Similarly, it will return the appropriate answer to the web chat (7) or the telegram (8), as the case may be. Finally, the answer is presented to the user via web chat (9) or Telegram (10).

### 3.2 Language recognition and automatic response

**Figure 2:**
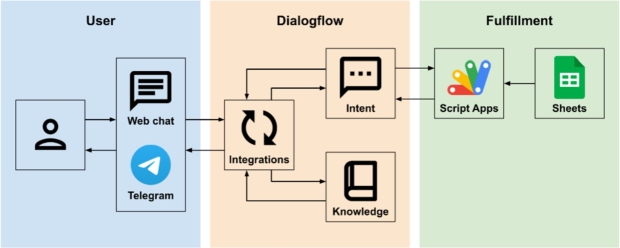
Components that connect to DialogFlow and are involved in the elaboration of the response.

When the assistant developed in DialogFlow receives a message from the student, it must provide a response to the student. The DialogFlow components used for language recognition and automatic response are *Knowledge Connectors* and *Intents* (Fig. 2).

#### 3.2.1 Knowledge connectors

A knowledge connector is a component within Dialogflow that enables the system to train itself by analysing different documents written in natural language. This method is particularly useful for incorporating multiple question-answer pairs, making it ideal for creating interactive FAQ websites. In this project, the Knowledge Connector used is based on a FAQ section from the official RIPEC website. By parsing the web page, Dialogflow extracts the question/answer pairs. A major advantage of this approach is the automated updating process. The system periodically scans the website for changes, ensuring that the knowledge base is kept up to date without any additional manual effort.

Knowledge connectors are useful when information can be structured in question-answer form, but they have limitations when it comes to providing tailored responses for each situation. To provide personalised answers and build more complex conversations, it is necessary to use another tool called *Intent*.

#### 3.2.2 Intents

**Figure 3:**
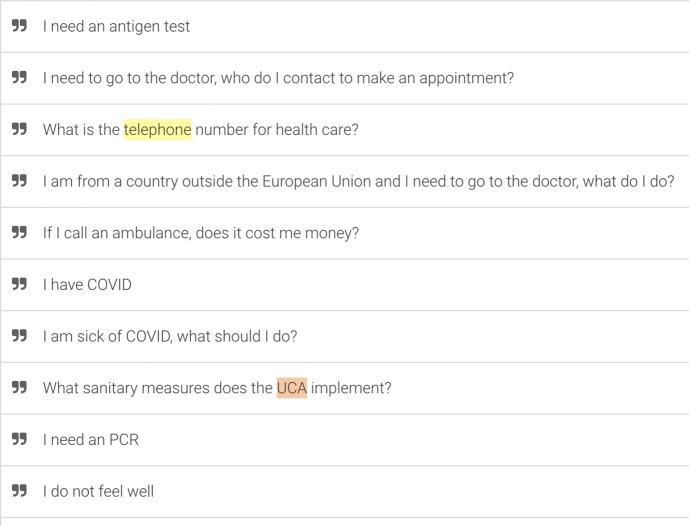
Training phrases for the Medical care intent.
• Parameters: the system can search for relevant data in interactions. Parameters are classified according to the type of entity they represent. DialogFlow provides many default entity types (e.g. cities, dates or numbers), but it is possible to create custom ones. For this chatbot, entities were created to recognise names of universities or specific data of the different services (e.g. phone numbers, emails or opening hours). In Fig. 3, you can also see how the parameters are highlighted in the training sentences.
• Responses: information sent back to the user. The extracted parameter values can be used within the responses to reassure the user that they have been captured correctly.

Intents are used to categorise user interactions so that responses can be tailored to each conversation. A more complex chatbot will have more intents. A basic Intent contains the following information:

• Training Phrases: A set of phrases that the user can use. It is unnecessary to include variants of the same phrase. However, if relevant parameters need to be extracted from interactions, it is useful to include examples of sentences in which they are used. In the Fig. 3, you can see the training phrases for the intent Medical Care.

In addition, the Intent includes some additional features to add complexity to conversations and responses. The following were used in this project

• Context: a structure that contains information relevant to contextualise the conversation. This chatbot collects in context the university where the student is studying or the student's country of origin.
• Fulfillment: functionality in Dialogflow that enables the integration of webhook services. When a message matches an Intent with Fulfillment activated, a request containing all the retrieved data is sent to the associated webhook service. A webhook is an HTTP callback that allows the NLP service to use a specific function defined in the webhook to make data processing more flexible [27]. The webhook service is responsible for processing the response based on the information available.

Twenty two intents have been implemented for this chatbot with the following names: Academic, Ask country, Ask service, Ask university, Default Fallback Intent, Add follow-up intent, Default Welcome Intent, Digital platform, Embassy information, Erasmus buddy program, Erasmus office, Goodbye, Housing, International airport, Library information, Local transport, Medical Care, Municipality, Psychological attention, Security, Service map, Student attention and, Visa service.

### 3.3 Webhooks and information management

Two Google services were used to create the webhooks for this chatbot: Google Apps Script and Google Drive.

#### 3.3.1 Google Apps Script

Apps Script is a platform for developing applications that can be easily integrated with the company's most popular services (such as Google Mail, Drive or Calendar). One of the most common uses of this service is to create macros to automate processes, but the same system also allows the creation of web applications. To handle the functionality of the chatbot, a service was necessary that could handle POST requests and respond with a JSON payload containing structured information. This information is critical for Dialogflow to process and generate appropriate responses for the bot user. The chatbot's programming language is primarily based on JavaScript, complemented by specific libraries tailored to interact with the various services involved in its operation. To implement the required behaviour, a function called doPost (a reserved name for this particular purpose) must be created, taking an input parameter to store the information from the POST request. The function should return a special type object called TextOutput, which contains the reply information for the request. Finally, the application needs to be deployed to make it operational and accessible.

#### 3.3.2 Google Drive

The decision to use Google Sheets as the storage platform for the bot's information was driven by several factors:

• Ease of use: Google Sheets provides a user-friendly interface that is familiar to most people. This was an advantage when staff from different universities involved in the project had to enter some of the information.
• Manageable amount of data: As the intention was not to handle a large amount of information, a complex storage system was unnecessary. Google Sheets provided a suitable solution for the project's needs.
• Seamless integration with Google Apps Script: Google Apps Script, which uses libraries that enable easy communication with Google Sheets, showed good compatibility with the spreadsheet service.

To ensure organised data management, the document consists of several sheets dedicated to storing information about countries, embassies, universities and the services they provide. In addition, to prevent errors, certain cells containing critical information essential to the operation of the system were locked. This ensures that these cells only accept a pre-defined set of values, guaranteeing consistent naming conventions for services and universities throughout the rows in which they are referenced.

It is the responsibility of each university's student services staff to complete their university's service information in this database so that the chatbot can respond to students with information specific to their university when they request it.

## 4 Evaluation

This evaluation will assess the chatbot to facilitate communication between student services offices and students in emergency situations in the future. For this purpose, firstly, some illustrative examples are introduces to show how the interactions between students and the chatbot look like. Secondly, the results of the evaluation carried out with users are shown.

### 4.1 Illustrative examples

**Figure 4:**
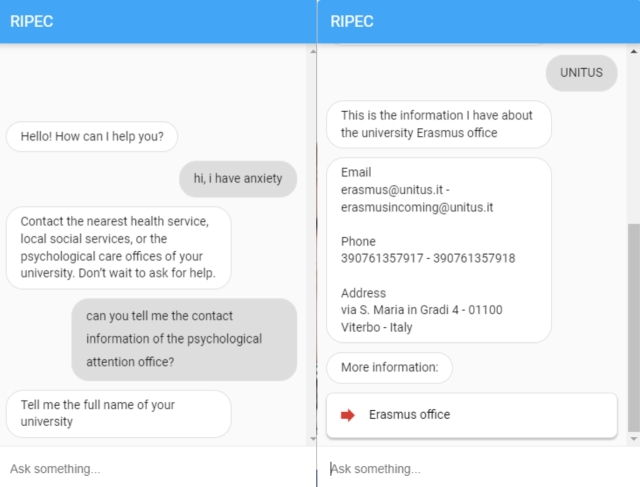
Illustrative Example 1 – conversation between the chatbot and a student using the chat within the website.

**Figure 5:**
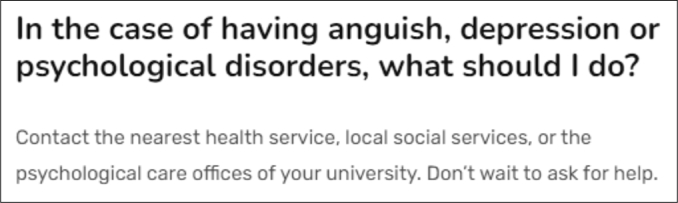
FAQ entry matching the student's question from use case 1.

In *Illustrative Example 1* (Fig. 4), we show a use of the chatbot from the chat integrated into the RIPEC project website. We have a student from the University of Tuscia (UNITUS) who is feeling anxious and is talking to the chatbot to see who he/she can contact. If you look at the screenshot on the left, you can see that the chatbot starts with a greeting and the student indicates that he/she is anxious. At this point, the DialogFlow assistant checks to see if there are any entries in its knowledge base that can be answered based on the information provided. It finds a related entry that matches the entry *“i feel anxious”* in the FAQ (Fig. 5). Based on the content of the FAQ, the chatbot recommends that the student contact the university's psychological support service.

The student then asks for the contact details of the psychological attention office. The chatbot does not find any FAQ entry that matches this question and tries to answer through the intents, matching the student's entry with the intent *Psychological attention*. The chatbot asks for the name of the university to establish this parameter, to set the context of the conversation and to direct the student to the specific services offered by his or her institution. The student indicates that his/her university is UNITUS. The chatbot then searches the database for information about UNITUS' psychological service and provides this information to the student.

**Figure 6:**
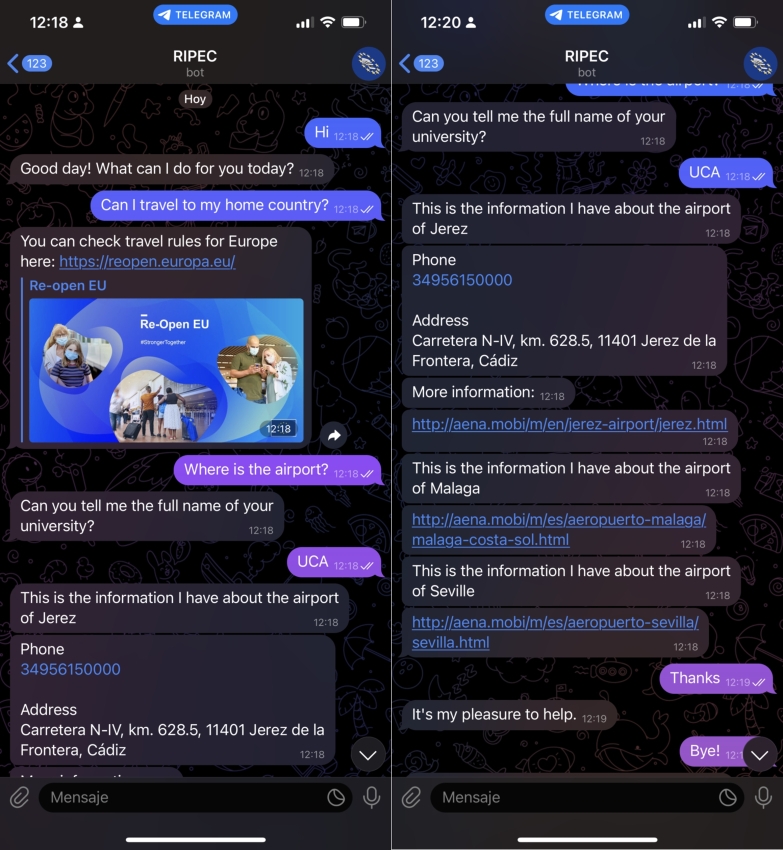
Illustrative Example – conversation between the chatbot and a student using the Telegram application.

**Figure 7:**
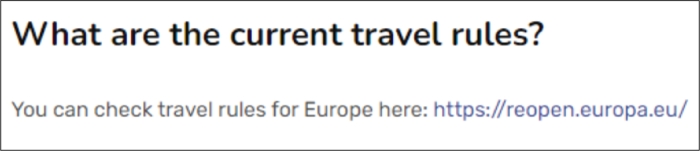
FAQ entry matching the student's question from use case 2.

In *Illustrative Example 2* (Fig. 6), we show the use of the chatbot through the Telegram messaging application. In this case, a foreign student studying at the University of Cádiz (UCA) wants to know if he/she can travel to his/her home country, so he/she asks the chatbot the question. The chatbot finds an entry in its knowledge base that matches the entry *“Can I travel to my home country?”* (Fig. 7) and invites the student to consult a web address.

After consulting the website, the student wants to know which airport he/she can fly from and asks the chatbot for this information. The chatbot has no knowledge base entry on this topic, so the intent *International airport* matches the question and the chatbot asks the student about his home university to provide the specific airport. The chatbot queries its database and returns to the student the airports registered as being close to the UCA.

### 4.2 Evaluation with users

This study was approved by the Ethics Committee in Non-Medical Experimentation and Genetically Modified Organisms of the University of Cádiz, approval number 005_2023. Additionally, informed consent was obtained from the participants in this research, whose participation was completely voluntary. The anonymity of the participants was maintained at all times.

This study adopts Oates et al.'s design and creation research strategy [28], in which we developed an IT artefact, specifically a chatbot, to investigate the perceptions of affected users in an academic context regarding the use of a chatbot in emergency situations. To evaluate the chatbot, a questionnaire was used as one of the accepted methods in this design and development strategy. For this purpose, the evaluation was carried out using the Bot Usability Scale (BUS) designed for chatbot users by Borsci et al. [29]. This questionnaire provides a list of attributes specifically developed to measure satisfaction with chatbots. The BUS questionnaire consists of 15 items divided into the following categories:

• Perceived accessibility (PA): Q01-Q02
• Perceived quality (PQ): Q03-Q09
• Perceived quality of conversation and information received (PC): Q10-Q13
• Perceived privacy and security (PPS): Q14
• Response time (TR): Q15

The items in the questionnaire are rated on a 5-point Likert scale, with 1 being the lowest (strongly disagree) and 5 being the highest (strongly agree).

The evaluation with users was carried out in two different scenarios.

#### 4.2.1 Scenario I: staff of the student services offices

Firstly, the chatbot was made available to the staff in charge of managing Erasmus students in 6 European universities: University of Tuscia (Italy), Scuola Superiore Carolina Albasio (Italy), Universidad de Cádiz (Spain), Panteio Panepistmio Koinonikon Kaipolitikon Epistimon (Greece), Wyzsza Szkola Kultury Spolecznej i Medialnej (Poland) and University Univerzita Jana Evangelisty Purkyne V Usti Nad Labem (Czech Republic). The evaluation of the chatbot was carried out as follows:

• Step 1: staff from these universities attended a presentation of the chatbot, which took place at one of the RIPEC project meetings.
• Step 2: after the meeting, they had two weeks to enter information from their universities into the chatbot database. Among the 6 universities, they created a total of 105 registers with services and information about their universities.
• Step 3: staff were asked to interact with the chatbot by posing different scenarios to it, and then to answer the BUS questionnaire. This evaluation was presented in an earlier paper [30] and is summarised in Table 1 for comparison with the students' evaluation.

**Table 1:** Survey, summary of results for items: identifier, category, question and average rating for staff and students.

Id	Cat.	Items	Staff	Students
Q01	PA	The chatbot function was easily detectable	4.00	3.65
Q02	PA	It was easy to find the chatbot	4.00	3.36
Q03	PQ	Communicating with the chatbot was clear	4.07	3.77
Q04	PQ	**Table:** I was immediately made aware of what information the chatbot can give me	3.85	3.55
Q05	PQ	**Table:** The interaction with the chatbot felt like an ongoing conversation	3.92	3.65
Q06	PQ	The chatbot was able to keep track of context	4.07	3.72
Q07	PQ	**Table:** The chatbot was able to make references to the website or service when appropriate	3.78	4.06
Q08	PQ	**Table:** The chatbot could handle situations in which the line of conversation was not clear	3.71	3.22
Q09	PQ	The chatbot's responses were easy to understand	4.50	3.99
Q10	PC	**Table:** I find that the chatbot understands what I want and helps me achieve my goal	3.92	3.67
Q11	PC	**Table:** The chatbot gives me the appropriate amount of information	3.85	3.76
Q12	PC	The chatbot only gives me the information I need	3.92	3.56
Q13	PC	I feel like the chatbot's responses were accurate	3.92	3.70
Q14	PPS	**Table:** The interaction with the chatbot felt secure in terms of privacy	4.51	3.50
Q15	TR	**Table:** My waiting time for a response from the chatbot was short	4.50	4.21

#### 4.2.2 Scenario II: students

Secondly, the chatbot was tested by 165 university students. The chatbot was introduced in several sessions to groups of 15 to 30 undergraduate and graduate students. All sessions followed the same structure:

• Step 1: the chatbot was introduced to the students and different use cases were shown, similar to those shown in Figure 4, Figure 6.
• Step 2: students had time to interact with the chatbot, resulting in a total of 405 interactions.
• Step 3: they completed the BUS questionnaire to evaluate the usefulness and usability of the chatbot.

#### 4.2.3 Results

The mean ratings of the staff and student evaluations of the BUS questionnaire are shown in the Table 1, columns four and five respectively.

In terms of perceived accessibility (PA), students' evaluation is above average, both in terms of the ease of recognising the functionality of the chatbot (3.65 for Q01) and the clarity of communication with the chatbot (3.36 for Q02). However, it is worth noting that the evaluation is slightly lower than that of the staff of the student services offices (4.00 for both Q01 and Q02).

In terms of perceived quality (PQ), students' evaluations are also above average for all items (Q03-Q09). The items that students rate most positively are the chatbot's ability to reference websites or services when appropriate (4.06 for Q07) and the ease of understanding the chatbot's responses (3.99 for Q09). In the case of Q07, it is worth noting that the students' evaluation was higher than that of the staff of the student services offices (3.78 for Q07).

Regarding the perceived quality of the conversation and the information provided, the students' evaluation is very balanced across the 4 items (Q10-Q13), with ratings ranging from 3.56 to 3.76. There is a generally positive evaluation from the student body, although it is also slightly lower than that of the staff of the student services offices.

In terms of privacy, the students' evaluation is one point lower than that of the staff of the student services offices (3.50 vs. 4.51 for Q14), although it is still above the average, this is an aspect to consider when implementing the chatbot.

Finally, the response time rating is very good (TR), with a rating of 4.21 (Q15), the highest average of all the students' items in the questionnaire.

#### 4.2.4 Validation

In order to check the validity of the survey, a Cronbach's alpha test was carried out on all the items as a whole, with a high level of confidence (0.94).

To test the significance of the data, we used the Chi-square test. As the data were collected using a 5-point Likert scale (BUS questionnaire), we will use the Stanine scale to organise the data. We will use a global variable that is the sum of all the ratings of the items of the BUS questionnaire and that reflects the acceptance of the chatbot by the students, i.e. chatbot acceptance.

Next, the validity of the results is analysed by looking at whether the chatbot acceptance is independent of gender, age, previous use of chatbots, being an Erasmus student and living away from home.

As can be seen in the Table 2, all p-values are greater than the significance value 0.05, so the null hypotheses that there is no relationship between student acceptance and respectively gender, age, having used chatbots previously, having been an Erasmus student and living away from home during the course cannot be rejected.

**Table 2:** Chatbot acceptance data.

	Gender	Age	Chatbot Use	Erasmus	Family Home
Chatbot acceptance	0.475	0.165	0.793	0.529	0.403

Then, we assessed the dependence of these same variables with respect to each of the questionnaire dimensions. If we look at the Table 3, we see that the p-value is always above the significance value 0.05, therefore, the null hypothesis cannot be accepted in any case, except in the relationship between gender and perceived accessibility.

**Table 3:** Data for questionnaire dimensions.

	Gender	Age	Chatbot Use	Erasmus	Family Home
PA	0.045	0.975	0.518	0.104	0.985
PQ	0.161	0.723	0.670	0.947	0.186
PC	0.297	0.168	0.439	0.649	0.191
PPS	0.690	0.462	0.392	0.535	0.099
TR	0.735	0.518	0.246	0.315	0.623

It is worth noting that students were able to give feedback on their impressions of the chatbot. This was collected via an open text field at the end of the questionnaire. Only 14 opinions were collected, but they were mostly positive. For instance, they highlighted its role as an intermediary that quickly and concisely directing you to any university website or phone number, and they highlighted the speed of the chatbot. In the area of improvements, they indicated that it would be important to disseminate it so that students could use it.

In this vein, we also analysed the students' evaluations in order to implement a chatbot that facilitates the life of university students beyond the management of emergency situations. This proposal received an evaluation of 4.17 out of 5 and, as can be seen in the Table 4, the results were also independent of the variables examined, i.e. gender, age, having used chatbots previously, having been an Erasmus student and living away from home during the course.

**Table 4:** Data chatbot implantation.

	Gender	Age	Chatbot Use	Erasmus	Family Home
Chatbot implantation	0.799	0.700	0.986	0.215	0.656

## 5 Discussion

In this paper, we propose the use of a chatbot to facilitate communication between students and student services offices in emergency situations, where protocols are evolving and users need to have up-to-date information. The tests conducted with the implemented chatbot and the evaluation conducted with users show a positive perception of the users involved, students and university staff, towards the usefulness of a chatbot for the intended purpose in the event of a new emergency situation.

The evaluation of the students has been positive in all dimensions, being always above average. This is the main objective, as they were the most affected when they were isolated by the confinement and subsequent measures due to the COVID-19 pandemic [31]. However, in almost all items, these ratings were slightly below the rating of the university staff. This is because the staff were part of the consortium in charge of developing the chatbot, so they are more knowledgeable about the problem.

On the other hand, the results have been validated regardless of whether students live away from home or not. Therefore, although the problem originates in the attention to this type of students, the data support that other students also detect the benefits of the chatbot and would consider using it if it were available.

The findings of this paper indicate that such chatbots can be used in future epidemics and emergency situations. It is the responsibility of universities to get involved and maintain this type of project so that, when the time comes, the university will be prepared to support its students [32], [33].

In conclusion, we consider it essential and therefore recommend implementing this type of solution in universities to improve communication and assistance to students, both in emergency situations and to obtain information on more common services of the institution. On many occasions, students complain about the difficulty of finding information within university websites and use Google instead of searching the university's own web pages. With a chatbot on the university's official website, students could be assisted in navigating institutional sites.

### 5.1 Threats to validity

Despite the positive results of our evaluations, it is important to recognise that the validity of our findings may be affected by certain factors.

• First, it should be noted that our study was conducted in a non-emergency setting. Therefore, it is necessary to test the chatbot in a real emergency context in order to fully assess its effectiveness and applicability.
• The chatbot requires maintenance by the universities. This is not a problem because using the chatbot is as simple as maintaining a FAQ and updating a spreadsheet-type database. However, university staff need to be convinced of its usefulness, and this can sometimes be an obstacle [34], [35].
• Although students do not provide information that identifies them, they do provide various pieces of information that could be considered quasi-identifying and that, all together, could be used to identify the person [36]. Although the chatbot developed does not collect logs, Google DialogFlow keeps a log for 400 days if you have logging enabled. And it is necessary to keep this log enabled for the chatbot to learn. Therefore, it would be advisable to consider an implementation in an open source tool that allows greater privacy and control by the university of this type of logs [37].

## 6 Conclusions

In universities, the use of chatbots has increased in recent years, as it allows teachers to facilitate students' autonomous work thanks to chatbot support. However, the use of these chatbots has been almost exclusively dedicated to teaching practice, and the COVID-19 pandemic and the subsequent measures to restore normality have shown that the technology has not covered all the needs of the student body.

This research proposes the use of a chatbot to provide information to students in emergency situations such as that experienced during COVID-19. The chatbot has been implemented and tested by students and student services staff, and the evaluation conducted shows positive evidence of the acceptance of the chatbot. The results show a positive perception of the effectiveness of chatbots in improving communication between the university and students, and their ability to respond quickly and accurately to users' questions and needs. The results also suggest that student services offices can use chatbots for more effective and dynamic communication with students, adaptable to changing regulations in emergency contexts. This type of tool would be particularly useful for students living away from home during their course, especially those outside their home country and unable to travel due to current government guidelines.

In future work, we suggest exploring the use of language models such as GPT to further improve the chatbot's ability to understand and respond to student questions more accurately and naturally, as well as implementing additional security measures to ensure user privacy.

## CRediT authorship contribution statement

Antonio Balderas: Conceived and designed the experiments; Performed the experiments; Analyzed and interpreted the data; Contributed reagents, materials, analysis tools or data; Wrote the paper.

Roberto Fermín García-Mena: Performed the experiments; Contributed reagents, materials, analysis tools or data.

Milagros Huerta: Conceived and designed the experiments; Performed the experiments; Analyzed and interpreted the data.

Néstor Mora: Conceived and designed the experiments; Performed the experiments; Analyzed and interpreted the data; Wrote the paper.

Juan Manuel Dodero: Analyzed and interpreted the data; Contributed reagents, materials, analysis tools or data; Wrote the paper.

## Declaration of Competing Interest

The authors declare the following financial interests/personal relationships which may be considered as potential competing interests:

Juan Manuel Dodero reports financial support was provided by Spanish National Research Agency (AEI), Grant PID2020-115844RB-I00. Nestor Mora reports financial support was provided by Erasmus+ Programme, Grant 2020-1-IT02-KA203-079711.

## Data Availability

Data will be made available on request.

## Appendix A Supplementary material

MMC The following survey aims to assess the usability and acceptance of the chatbot developed within the RIPEC project. MMC

## Footnotes

1 https://github.com/RIPEC/Dialogflow/wiki.
