# Supplementary material for: Chatbot for communicating with university students in emergency situation
Source: Heliyon. 2023 Aug 30;9(9):e19517. doi: 10.1016/j.heliyon.2023.e19517 (PMC10558753; doi:10.1016/j.heliyon.2023.e19517)
Supplement: MMC — The following survey aims to assess the usability and acceptance of the chatbot developed within the RIPEC project. [file mmc1.docx]

# Chatbot for Communicating with University Students in Emergency Situation

The following survey aims to assess the usability and acceptance of the chatbot developed within the RIPEC project.

## Section 1: Respondent data

|  | Female | Male | I prefer not to answer | Other |
| --- | --- | --- | --- | --- |
| Gender |  |  |  |  |

Age _______________________

Degree ________________________

|  | Yes | No |
| --- | --- | --- |
| During the academic year, do you live in your usual family home? |  |  |

|  | Yes | Not now, but in previous years | No |
| --- | --- | --- | --- |
| Are you or have you been an Erasmus student? |  |  |  |

| Please check a value from 1 to 5, considering that 1 means "not at all" and 5 means "very familiar" | | | | | |
| --- | --- | --- | --- | --- | --- |
|  | 1 | 2 | 3 | 4 | 5 |
| Are you familiar with chatbots? |  |  |  |  |  |

| Please check a value from 1 to 5, considering that 1 means "not at all" and 5 means "very often" | | | | | |
| --- | --- | --- | --- | --- | --- |
|  | 1 | 2 | 3 | 4 | 5 |
| Have you interacted with chatbots? |  |  |  |  |  |

| Please check a value from 1 to 5, where 1 means "never" and 5 means "daily" | | | | | |
| --- | --- | --- | --- | --- | --- |
|  | 1 | 2 | 3 | 4 | 5 |
| How often do you interact with chatbots on a weekly basis? |  |  |  |  |  |

## Section 2: RIPEC Project Chatbot Questionnaire

For all the items: please check a value from 1 to 5, where 1 means " Strongly disagree " and 5 means " Strongly agree "

**Perception of the accessibility of the functions of the chatbot**

|  |  | 1 | 2 | 3 | 4 | 5 |
| --- | --- | --- | --- | --- | --- | --- |
| Q01 | The functionality of the chatbot is easily detectable |  |  |  |  |  |
| Q02 | It is easy to find the chatbot |  |  |  |  |  |

**Perceived quality of chatbot functions**

|  |  | 1 | 2 | 3 | 4 | 5 |
| --- | --- | --- | --- | --- | --- | --- |
| Q03 | Communication with the chatbot is clear |  |  |  |  |  |
| Q04 | I was immediately made aware of what information the chatbot can give me |  |  |  |  |  |
| Q05 | The interaction with the chatbot felt like an ongoing conversation |  |  |  |  |  |
| Q06 | The chatbot was able to keep track of context |  |  |  |  |  |
| Q07 | The chatbot was able to make references to the website or service when appropriate |  |  |  |  |  |
| Q08 | The chatbot could handle situations in which the line of conversation was not clear |  |  |  |  |  |
| Q09 | The chatbot’s responses were easy to understand |  |  |  |  |  |

**Perceived quality of conversation and information provided**

|  |  | 1 | 2 | 3 | 4 | 5 |
| --- | --- | --- | --- | --- | --- | --- |
| Q10 | I find that the chatbot understands what I want and helps me achieve my goal |  |  |  |  |  |
| Q11 | The chatbot gives me the appropriate amount of information |  |  |  |  |  |
| Q12 | The chatbot only gives me the information I need |  |  |  |  |  |
| Q13 | I feel like the chatbot’s responses were accurate |  |  |  |  |  |

**Perceived privacy and security**

|  |  | 1 | 2 | 3 | 4 | 5 |
| --- | --- | --- | --- | --- | --- | --- |
| Q14 | The interaction with the chatbot felt secure in terms of privacy |  |  |  |  |  |

**Time response**

|  |  | 1 | 2 | 3 | 4 | 5 |
| --- | --- | --- | --- | --- | --- | --- |
| Q15 | My waiting time for a response from the chatbot was short |  |  |  |  |  |
